# The Health Impacts of Air Pollution in the Context of Changing Climate in Africa: A Narrative Review with Recommendations for Action

**DOI:** 10.5334/aogh.4527

**Published:** 2024-12-05

**Authors:** Lynn M Atuyambe, Raphael E Arku, Natasha Naidoo, Thandi Kapwata, Kwaku Poku Asante, Guéladio Cissé, Belay Simane, Caradee Y Wright, Kiros Berhane

**Affiliations:** 1 Makerere University, School of Public Health, Uganda; 2 The Eastern Africa GEOHealth HUB, Uganda; 3 Department of Environmental Health Sciences, School of Public Health and Health Sciences, University of Massachusetts Amherst, USA; 4 Environment and Health Research Unit, South African Medical Research Council, Pretoria, South Africa; 5 Department of Environmental Health, Faculty of Health Sciences, University of Johannesburg, Johannesburg, South Africa; 6 Kintampo Health Research Centre, Research and Development Division, Ghana Health Service, Kintampo North Municipality, Ghana; 7 Swiss Tropical and Public Health Institute, Basel, Switzerland; 8 University of Basel, Basel, Switzerland; 9 Addis Ababa University, Addis Ababa, Ethiopia; 10 Columbia University, New York, USA

**Keywords:** Adaptation, air pollution, fossil fuel burning, energy, environmental health, vulnerability, particulate matter, PM_2.5_

## Abstract

*Introduction:* Despite the broad improvement in air quality, air pollution remains a major leading global risk factor for ill health and deaths each year. Air pollution has a significant impact on both health and economic growth in Africa. This paper reviews the health impacts of air pollution and the benefits of air pollution mitigation and prevention on climate change.

*Methods:* We conducted a narrative review and synthesized current literature on the health impact of air pollution in the context of changing climate in Africa.

*Results:* Particulate matter (PM_2.5_) concentrations in Africa pose significant health risks due to various sources, including household fuels and industrial emissions. Limited air quality monitoring hampers accurate assessment and public health planning. Africa’s rapid urbanization exacerbates air pollution, impacting vulnerable populations disproportionately. Renewable energy adoption and improved monitoring infrastructure are crucial for mitigating air pollution’s economic and health impacts. Recommendations include adopting air quality standards, identifying pollution sources, and prioritizing interventions for vulnerable groups. Integrating renewable energy into development plans is essential for sustainable growth. African leaders must prioritize environmental policies to safeguard public health amid ongoing industrialization.

*Conclusions:* Air pollution prevention remains a vital concern that requires leaders to engage stakeholders, and other opinion leaders in society. African leaders should proactively explore new avenues to integrate non‑polluting renewable energy sources such as solar power, wind and hydropower into their national development plans.

## Introduction

In parts of the global north, significant efforts by local authorities have led to declining air pollution levels over the years, resulting in health improvements [1–4]. However, air pollutant levels are detrimentally high in low‑ and middle‑income countries (LMICs), especially in Asia and Africa [3]. For instance, a global assessment of fine particulate matter (PM_2.5_) levels suggests that five out of the ten most heavily polluted countries in the world are in Africa [5]. In most African countries, increasing population growth, rapid urbanization, expanding economic activities and the demand for energy are contributing substantially to the worsening of air quality in the region. Air pollution in the region is also shown to be negatively impacted by global climate change.

The strong linkages and interconnectedness between climate change and air pollution have been well established [6]. Global warming leads to increasing emissions of some air pollutants, while other air pollutants exacerbate climate change [6]. Future projections of extreme weather events, including prolonged and extreme temperatures and drought on the continent, are expected to worsen the already poor air quality in Africa. Drought‑induced dust storms are becoming more common [7], while excessive urban heat necessitates more air conditioning and the use of diesel generators in cities [8]. Sustained biomass fuel use in both rural and urban areas remains an important source of household energy [9]. Both diesel generators and biomass stoves [10] are known sources of black carbon pollution, which is a combustion‑related component of PM_2.5_ and a contributor to global climate change [11]. Consequently, efforts to address climate change will simultaneously influence air quality and vice versa.

Despite the broad improvement in air quality globally over the last few decades, air pollution remains a major leading risk factor for ill health and contributes to millions of deaths each year. It is considered the single largest preventable environmental risk factor for illness and deaths. It affects nearly everyone and almost every organ in the body [12]. A large body of epidemiological studies have documented links between air pollution and a wide variety of adverse health outcomes, emphasizing the considerable role of air pollution in the general population’s disease burden and mortality risk [13]. Air pollutants contribute to the onset of allergies and asthma amongst other chronic respiratory and cardiovascular diseases. In 2019 alone, 4.2 million people were estimated to have died worldwide from PM_2.5_ exposure and the death toll from PM_2.5_ surpassed that of other air pollutants [14, 15]. In recognition of its devastating impacts, the World Health Organization (WHO) recently issued more stringent air quality guidelines to spur on further action on reducing emissions [16].

Africa is amongst the world’s regions with the highest air pollution related health and economic consequences, commensurate with high and widely varied exposure levels [17]. In 2019, for instance, air pollution was the second leading risk factor for death across Africa, with the death rate almost double the global average [5]. As the economic conditions of countries in Africa improve amidst high population growth and high rate of urbanization, the continent will continue to experience some of the worst air quality and the most severe health consequences. Thus, Africa is presently grappling with the challenge of reducing air pollution emissions and exposures and their attributable health impacts, and at the same time, she must take steps to mitigate the role of climate change. Addressing such a challenge and the complexity of air pollution in a changing climate requires multi‑stakeholder engagement and multi‑sectorial approaches. A particular focus needs to be placed on reducing exposure to PM_2.5_ pollution and its components due to its disproportionate impact on human health, without ignoring the rising emission levels of combustions related pollutants like nitrogen dioxide (NO_2_) in growing cities in Sub‑Saharan Africa (SSA) [18].

In this review, we focus on PM_2.5_ pollution, which is the most extensively studied and the best evidence‑based indicator of the health effects of pollutants [19]. When inhaled, PM_2.5_ can reach deep into the lungs and even into the bloodstream, causing adverse health effects such as reduced lung function and growth in children, chronic heart diseases, chronic obstructive lung diseases and premature death in adults [20]. It is also the pollutant that is most likely to be impacted by climate change [20]. Here, we employ narrative review methods to offer comprehensive analysis of the health consequences of air pollution in Africa. We synthesize the evidence about adverse health outcomes due to rising levels of ambient and indoor PM_2.5_ in the face of climate change. The paper also examines the co‑benefits of air pollution mitigation and prevention of climate change to allow policymakers and researchers gain a more holistic understanding of the situation for effective multi‑disciplinary approaches to enable decision‑making processes. Finally, we propose actionable recommendations that, when implemented, could protect vulnerable populations in Africa. These actions may also be applicable in other resource‑constrained countries outside the continent.

## Methodology

### Study design

We conducted a narrative review and synthesized current literature on the health impacts of air pollution in the context of a changing climate in Africa. Our goal is to present a review of evidence to inform policy and practice and identify potential gaps in need of further research [21, 22]. We relied on relevant studies, reviews, and reports published in the last five years on human health effects related to ambient and household air pollution, with a focus on PM_2.5_ pollution in Africa. This period was chosen since earlier reviews [23–25] were published between 2018 and 2021, and we aimed to further synthesize the most recent evidence. This review is part of the ‘Future of Health and Economic Resilience in Africa’ (FHERA) project (https://www.hsph.harvard.edu/fhera/)[26] – a collaboration between *The Lancet* and a core panel of experts and stakeholders from across Africa and beyond, primarily housed at the Harvard TH Chan School of Public Health.

Our approach followed the Population, Exposure, Concept / Context, Outcome and Study (PECOS) [27] framework and identified studies eligible for inclusion using the following criteria:

- *Population*: The targeted population was specific to Africa. Special attention was given to household sources of air pollution and vulnerable groups.
- *Exposure*: Studies that investigated PM_2.5_ exposure and its health effects.
- *Concept/Context*: Literature review on PM_2.5_ and its health impacts in Africa.
- *Outcome*: Health outcomes related to PM_2.5_ exposure including morbidity and mortality.
- *Study designs*: Primary quantitative and qualitative studies that have investigated health effects due to air pollution.

### Search strategy

We first conducted a pilot search strategy to enhance our search precision. A comprehensive literature search was then conducted after the pilot search in five relevant electronic databases: Web of Science Core Collection (accessed via Web of Science), Scopus (accessed via Elsevier), CAB Abstracts (accessed via Web of Science/OVID), MEDLINE and EMBASE. Additionally, the reference lists of eligible articles were manually searched for more articles relevant to the narrative review. The databases were then searched using search terms agreed upon by the authors for air pollution and human health impacts from 31 July 2019 until 31 July 2023 to make the review post in the IPCC‑6 report [28].

The keywords included were those related to:

- *Exposures*: air pollution, air quality, dust storms or windblown dust, actions on clean air quality, air quality standards, indoor air quality, household air pollution, indoor air pollution, etc.
- *Outcomes*: health effects, health impacts, mortality, morbidity, diet, nutrition, mobility, injury, and mental health, neurodevelopment, etc.

The database searches were supplemented with materials in the grey literature, including the websites of the African Development Bank, International Development Research Centre, United Kingdom Department for International Development, United National Environment Programme, World Health Organization, World Meteorological Organization, World Bank, United Nations, United Nations Educational, Scientific and Cultural Organization (UNESCO), Intergovernmental Panel on Climate Change, United Nations Framework for the Convention on Climate Change (UNFCCC), and theses and dissertations. No content was excluded from the grey literature category, regardless of publication date, language or author’s country of origin, as long as the content pertains to Africa. Results of the searches were managed in Mendeley^TM^.

## Results and Discussion

### Conceptual framework

**Figure 1:**
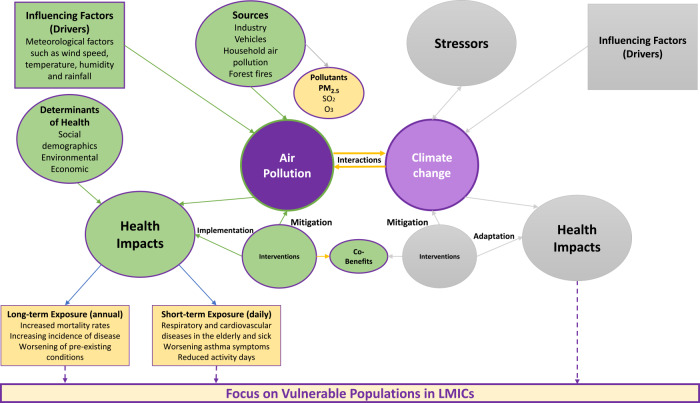
Conceptual framework illustrating the determinants and impacts of air pollution and the interactions between air pollution and climate change. The diagram illustrates the linkages between air pollution and climate change. In the centre, yellow arrows represent the connections between air pollution and climate change (shown in purple). The left side of the diagram focuses on air pollution‑related factors, including its primary sources (top left: green and yellow circles), the health impacts of pollution (green circle) and climate‑related variables influencing air pollution (green box). Targeted interventions (green circle) that address both air pollution and climate change offer co‑benefits across sectors such as health, environment, and socioeconomic development. Vulnerable populations in LMICs, positioned at the foundation of the diagram, are central to understanding these relationships. The right side of the diagram, associated with climate change, is greyed out, as it is beyond the scope of this review.

The interplay between the determinants of air pollution and subsequent impact on human health and well‑being, with attention to vulnerable populations, as well as the potential interactions and co‑benefits between air pollution and climate change, is illustrated in Figure 1. Air pollution and climate change have been demonstrated to have several interactions in terms of sources, emissions and health consequences with feedback loops [29]. Certain air pollutants, such as black carbon (BC) and methane, can contribute to climate change by absorbing sunlight and warming the atmosphere [30]. Air pollutants, including nitrogen oxides (NOx) and volatile organic compounds (VOCs), react in the presence of sunlight to form ground‑level ozone, a harmful air pollutant and greenhouse gas [31]. Climate change can, in turn, exacerbate certain forms of air pollution. Higher temperatures can lead to the formation of ground‑level ozone and smog and contribute to PM formation, with adverse impacts on human health. Changes in temperature and extreme weather events will likely influence the air pollutant mixture and patterns and pose health risk to all, especially amongst disadvantaged and vulnerable populations [32–34]. Climate change modifies the availability and distribution of plant‑ and fungal‑derived allergens and increases the frequency of extreme climate events [35] such as droughts, wildfires, dust storms and coastal flooding [36, 37], all with major and varied implications for air quality. Both air pollution and climate change have direct and indirect effects on human health [29]. Poor air quality can cause or exacerbate respiratory and cardiovascular diseases, while climate change can intensify the spread of certain diseases and impact health through major effect on food systems, extreme weather events such as heatwaves, droughts and floods, and changes in the distribution of allergens [38].

### PM_2.5_ concentrations in africa

Particulate matter (PM) is made up of a complex mixture of suspended gases and other contaminants [24], solid particles and liquid droplets in the air [39]. PM_2.5_ is the most critical air pollutant due to its known associated health risks and adverse health outcomes in exposed populations [40]. It is most commonly associated with cardiovascular and respiratory diseases and deaths [40]. PM_2.5_ emitted directly into the atmosphere come from both natural (e.g. dust storms and forest fires) and man‑made (e.g. fossil or biomass fuel combustion and cigarette smoke) sources [41]. Secondary PM_2.5_ particles come from chemical reactions occurring between particles from both anthropogenic and natural sources [41]. Some emission sources of PM_2.5_ pollution in Africa are similar to those in high‑income countries, such as transportation and industry. PM_2.5_ pollution in Africa also has some unique features, including biomass fuels for household and commercial activities, kerosene and diesel generator use for lighting, and trash and agricultural burning practices. Based on global satellite data, Africa experiences the most frequent fire occurrences [40], which is a known source of PM. Other sources of PM_2.5_ in Africa encompass household, commercial and industrial use of coal as well as periodic dust blown from the Sahara Desert [40].

Presently, many cities lack ground‑based measurement data for accurate air quality and health and climate impact assessments [42]. In SSA, the average distance from one air quality monitor to the other amounts to roughly 500 km (310 miles). In Central Africa, it exceeds 1000 km (>600 miles) [42]. This considerable distance underscores the challenge of accurately representing local air quality conditions through existing monitoring infrastructure [42]. In 2020, the air quality monitoring network in Africa expanded to include five new countries (Senegal, Mali, Ivory Coast, Madagascar and Kenya) and ten new cities [40]. Forty‑one African countries lack air quality monitoring data, leaving nearly a billion people without the necessary information for public health planning and management [40].

Africa’s population is anticipated to undergo a two‑fold increase within the next three decades, posing challenges in effectively managing air quality and ensuring clean air amid the ongoing rapid urban and economic growth [40]. In particular, cities in SSA are in economic transition and undergoing significant expansion, and consequently are experiencing high levels of PM_2.5_ pollution from diverse sources. Technological advancement and the emergence of low‑cost sensors promise to improve data scarcity in growing African cities. Yet, relative to other regions of the world, Africa is still doing poorly in terms of data generation from low‑cost sensors. Out of the ~1700 cities worldwide with average populations of at least 300,000, there are only approximately 5500 low‑cost ground‑based PM_2.5_ monitors available, with more than half of those monitors located in China or the United States [42]. Although the majority of air pollution‑related deaths (>85%) and health burden are estimated to occur in LMICs, including those in Africa [43], the density of air quality monitoring is notably lower in Africa than in any other world region, averaging only 0.03 monitor density per million inhabitants [42]. This level of monitoring and data availability is insufficient for effectively managing air quality for the ~1.2 billion African population [42].

Obtaining more accurate assessments of the spatial and temporal dispersion of PM_2.5_ levels will identify areas of significant concern, monitor progress and provide valuable insights for local air quality management planning [42, 44, 45]. Expanding air quality monitoring across Africa is now necessary for generating the needed data for making informed decisions about pollution and public health on the continent. Low‑cost PM sensors are being promoted to improve air quality data availability in the region [46]. A large network of low‑cost sensors will help characterize and monitor intra‑ and inter‑urban inequalities of fine particle pollution exposures over space and time, particularly in complex pollution‑source areas. It is hoped that widely available community‑level data would build public demand for clean air, which will in turn drive policy action on air pollution in Africa. However, careful consideration of the local context and data quality and validity is necessary to collect high‑quality data in cost‑effective and efficient ways while using low‑cost sensors [47]. Even though low‑cost sensors are promising as means to solving data scarcity in Africa, there are major concerns about the accuracy, precision and overall quality of the data being generated [47]. Thus, using such small, affordable air quality sensors will require comprehensive evaluation and calibrations to ensure that end‑users are informed about sensor performance capabilities and limitations [48].

Country specific air quality management plans and policies should also consider the broader geographical context. For instance, ambient PM_2.5_ trends suggest that countries with lower levels of sociodemographic development tend to experience higher PM_2.5_ exposure [49]. This is largely true for countries in SSA, which generally have the lowest economic power and exhibit the highest levels of PM_2.5_ [49]. However, the observed correlation is flawed by the fact that PM_2.5_ is a regional pollutant and can be carried long distances and affect neighbouring countries regardless of their levels of development [49]. Consequently, some of the higher income countries in North Africa may experience elevated levels of PM_2.5_ pollution due to both local sources and transported/regional dust storms [49]. In North Africa, non‑industrial activity accounted for the largest share of PM_2.5_ emissions in the past decades, comprising 38.2% of total emissions, followed by road traffic at 21.5% and other industrial combustion at 17.3% [50]. From 1990 to 2015, PM_2.5_ in the five North African countries significantly exceeded the current WHO annual Air Quality Guideline (AQG) of 5 µg/m^3^ [50, 51].

**Figure 2:**
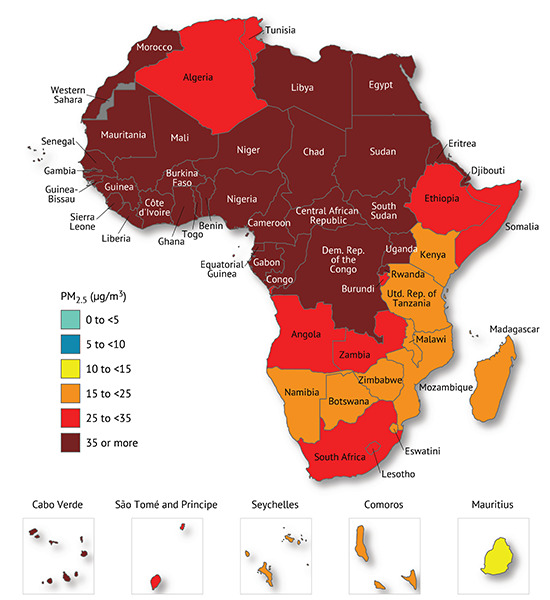
The population‑weighted annual average PM_2.5_ exposures in the African sub regions [40].

In Africa, the entire population is estimated to reside in regions where annual PM_2.5_ levels exceed the currently revised WHO AQG of 5 μg/m^3^ (Figure 2). In 2019, countries with the highest overall annual mean PM_2.5_ pollution in Africa included Niger (80.1 μg/m^3^), Nigeria (70.4 μg/m^3^), Egypt (~67.9 μg/m^3^), Mauritania (66.8 μg/m^3^) and Cameroon (65.5 μg/m^3^) [49]. Several other countries experienced levels above 35 μg/m^3^, the least stringent WHO interim target [49]. In terms of subregional variations, Western Africa has the highest PM_2.5_ pollution levels, with an average concentration of 64 μg/m^3^, while Southern Africa has the lowest at about 27 μg/m^3^ [49]. However, there are important between‑country differences within each subregional block. A large share of residents in some countries in Southern Africa, such as Namibia, Zimbabwe and Tanzania, reside in areas that only meet the WHO’s least stringent interim target of 35 μg/m^3^ [49]. Conversely, in Western Africa, over 90% of the population live in areas where the PM_2.5_ levels do not meet even the least stringent interim target [49]. In each country, additionally, there are major urban‑rural disparities in PM_2.5_ concentration, with the highest levels concentrated in urban areas [49]. All ten of the most populous cities in Africa experience PM_2.5_ levels above the WHO annual AQG, and four of these cities surpass even the least stringent interim target [49]. Additionally, within‑city exposure disparities are also of major concern. Fine spatial and temporal data from Accra, one of the fastest growing metropolises in West Africa, show large variations in land use and neighbourhood features [16, 52].

**Figure 3:**
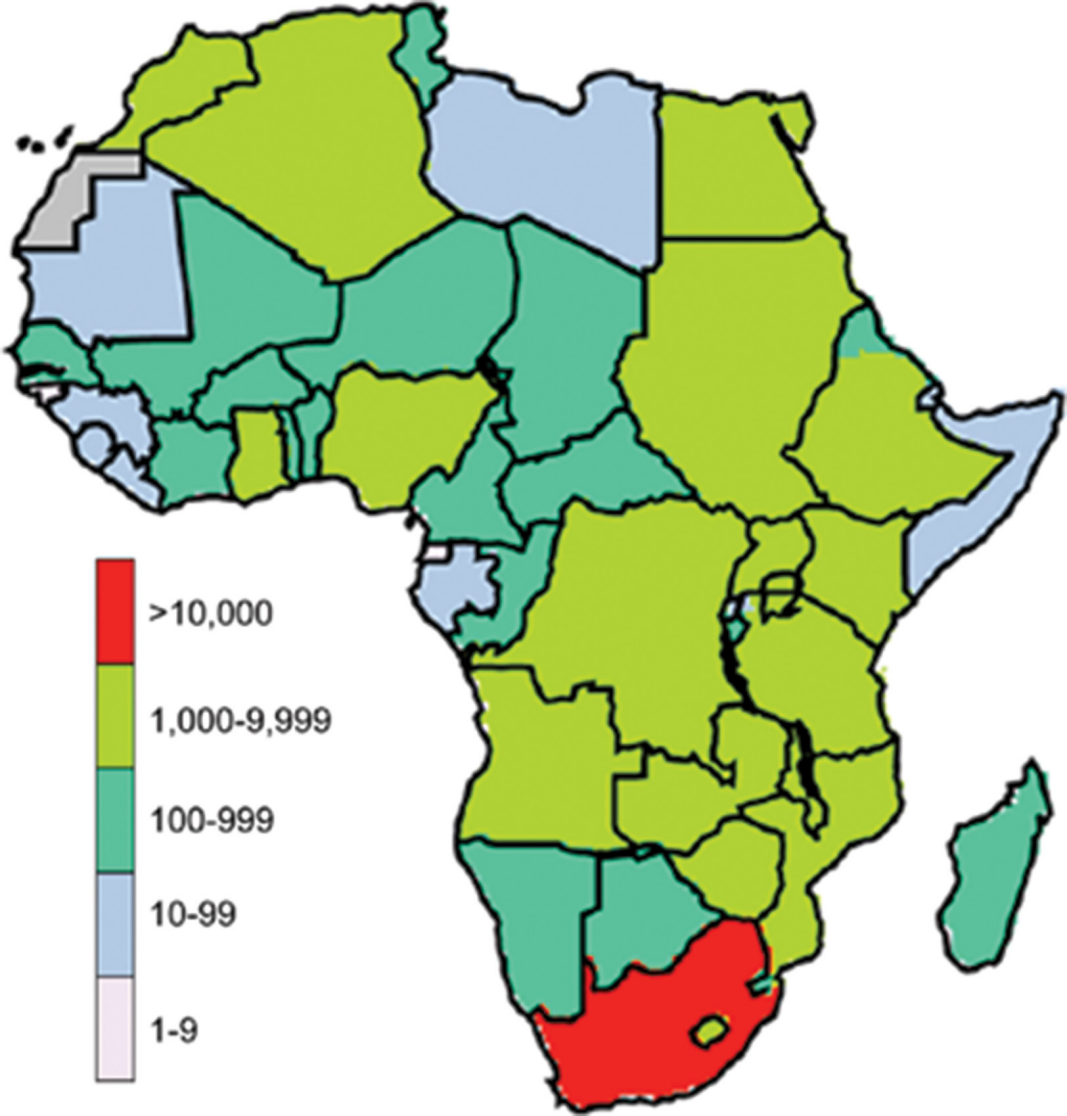
Impact of increased fossil fuel consumption on mortality: additional premature deaths in African countries due to PM_2.5_ exposure in 2030 compared with 2012 [45].

Using the GEOS‑Chem model, a recent study simulated PM_2.5_ levels across Africa to assess the health burden of future fossil fuel use in terms of excess deaths (Figure 3) [46]. The model prediction showed that there would be 48,000 avoidable deaths by 2030, attributable to fossil fuel emissions from power plants and transport, with the majority of these deaths projected to occur in South Africa (10,400), Nigeria (7500) and Malawi (2400). Estimates for excess mortality rates from power plants would be three times higher than those from traffic related sources [46]. The differential impact of air pollution on the burden of disease is influenced by population size in relation to air quality across various regions, highlighting that strategies aimed at reducing emissions would be more effective in some places than others [46].

In another study, the annual PM_2.5_ concentrations of several African countries were analysed using low‑cost sensors [53]. Although low‑cost sensors generally tend to underestimate particle concentrations at locations with high pollution, temperature and relative humidity conditions, the results provide insight into PM_2.5_ pollution across the 15 monitoring sites in 11 cities in eight Sub‑Saharan African countries. The annual PM_2.5_ concentrations across sites ranged from as low as 10 µg/m^3^ in the Gambia to as high as 116 µg/m^3^ in Cameroon, with every site surpassing the current WHO annual AQG of 5 µg/m^3^ [53]. Compared with the East African subregion, the West African subregion experiences periodic Harmattan dust storms from the Sahara Desert which produce major substantial seasonal difference in PM_2.5_ pollution, where the levels can be two to five times higher than in the non‑harmattan period [53–55].

### Economic impacts of health effects of air pollution in africa

The economic impact of diseases associated with air pollution is profound, accounting for 6.5% of Africa’s Gross Domestic Product (GDP) annually in healthcare costs [49]. In 2019, the Global Burden of Disease project showed that Egypt, Ghana, the Democratic Republic of Congo, Kenya and South Africa altogether spent more than US$5.4 billion on managing the health impacts caused by air pollution [56]. While this amount may sound little relative to the combined gross dometic product (GDP) of these five countries [25], it does not account for the broader economic consequences of air pollution on productivity and quality of life. A more accurate and detailed analysis that considers a wider range of economic indicators, such as social and environmental implications of air pollution, is needed to fully understand the impact of air pollution on Africa’s economy and well‑being. This estimation may assist policymakers in the prioritization of interventions to minimize air pollution, long term planning of mitigation strategies and advocacy efforts [57].

### Population growth, urbanization, industrialization and air pollution in africa

Africa is currently experiencing the most rapid population growth compared with the rest of the world. Africa’s population is projected to surpass two billion by 2050 with nearly 60% expected to reside in urban areas compared to less than 40% in 2011 [58]. Rapid urban growth and economic expansion, combined with increasing industrial activities, greater vehicle ownership and continued reliance on biomass for domestic energy, has the potential to significantly deteriorate air quality across the continent [58]. The lack of strong environmental policies and the relatively weak enforcement mechanism to curb the importation of used older polluting vehicles in several African countries, as well as the poor regulation of vehicle emissions also means more traffic related emissions which contribute to rising urban air pollution [59–61]. Fewer vehicles or a significant shift to low emission vehicles would significantly reduce urban air pollution. However, many African cities lack the resources for reliable and low‑emission mass transport systems.

### Association of PM_2.5_ with adverse health outcomes in africa

#### Long‑term and short‑term exposure to PM_2.5_

Epidemiological studies have evaluated the links between long‑term or short‑term PM_2.5_ exposures and adverse health outcomes [19]. The short‑term exposure window refers to periods of hours to days, usually examined through time‑series study designs, where health outcomes (e,g. mortality rates or hospital admissions) vary with day‑to‑day fluctuations in PM_2.5_ concentrations. The segment of the population with pre‑existing diseases, such as respiratory or cardiovascular conditions, is more sensitive to short‑term PM_2.5_ exposures. Peaks of elevated PM_2.5_ concentrations can trigger acute health effects or exacerbate existing health conditions in susceptible individuals, including infants and children, pregnant women, the elderly, and people with pre‑existing diseases [19]. Long‑term exposures refer to periods of months to years and are typically assessed through cohort studies that investigate the associations between spatial variations in long‑term average concentrations of PM_2.5_ and health outcomes. Long‑term exposure to PM_2.5_ has the potential to affect the entire population by contributing to the initiation or progression of various diseases like respiratory and cardiovascular disorders. However, the health effects of long‑term exposure are less immediately noticeable than those of short‑term exposure.

#### Health outcomes associated with ambient PM_2.5_ in Africa

Poor air quality in fast‑growing African cities is implicated in declining birth rates, shorter life expectancy and a shift from traditional health threats like infectious diseases and malnutrition to chronic non‑communicable ailments such as hypertension, heart disease and diabetes [58]. Although chronic obstructive pulmonary disease (COPD) is considered the overall leading cause of death linked to PM_2.5_ pollution in Africa, diabetes and heart disease tops in five African countries with the highest PM_2.5_ levels [49]. Other leading causes of death linked to air pollution include stroke, lung cancer and lower respiratory infections [49]. Short‑term studies showed greater risks for respiratory diseases (mostly in children), whereas long‑term studies exhibited higher risks for cardiovascular diseases (mostly in adults) [62].

In a recent review of 23 studies assessing the health effects of air pollution in Africa, it was observed that South Africa contributed the most to the research, while most countries in the SSA region severely lacked data. The lack of epidemiologic research in the context of SSA is concerning and could be most likely due to the paucity of available continuous monitoring networks (exposure data) in the region [63]. The few available studies on health effects associated with air pollution were primarily focused on self‑reported respiratory symptoms, which indicates a limited scope of investigation [63]. However, those findings highlighted the heightened vulnerability of children and the elderly to the detrimental impacts of air pollution [63]. Consequently, it becomes crucial to establish and institutionalize robust air pollution monitoring systems in African cities, identify the primary sources of pollution and formulate and enforce legislative measures to reduce emissions and mitigate the adverse health effects associated with air pollution [63].

#### Health outcomes associated with household sources of PM_2.5_ concentrations

Household PM_2.5_ concentrations caused by the inefficient combustion of polluting fuels such as wood, charcoal, coal, crop residues, and kerosene for cooking, heating, and lighting, is another significant contributor to ill health and deaths in Africa and considered one of the most prominent environmental risks to public health. Exposure to household PM_2.5_ is responsible for an estimated 2.3 (range 1.6–3.1 million) million premature deaths worldwide annually [64]. Children are thought to bear the highest burden of the health effects of household air pollution. Despite a one‑third reduction in under‑five mortality attributable to household air pollution between 2000 and 2017, the negative health effects persist across the continent [65]. Household air pollution sources also permeate the outdoor environment and emerge as major contributors to ambient PM_2.5_ pollution [64]. In 2019, PM_2.5_ pollution, encompassing both ambient and household PM_2.5_, emerged as the primary risk factor for fatalities across the continent [49].

Within rural households, the average concentration of PM_2.5_ in the primary cooking area is estimated to exceed 500 μg/m³ [66]. For example, in Ethiopia, the average PM_2.5_ concentration in indoor cooking areas surpassed 1200 μg/m³, which is 240 times the new WHO guideline of 5 μg/m³ [51]. The reliance on solid fuels for cooking is prevalent in several African countries. The Central African Republic, South Sudan, Rwanda, Burundi, Niger, Mali, Madagascar, Tanzania, Uganda and Guinea‑Bissau are the top ten countries with the highest share of households relying on solid fuels for cooking [49]. In each of these countries, over 97% of the population uses solid fuels as their primary cooking source [49]. During the period 2010–2019, despite a steady decline in the use of solid fuels for cooking, numerous African nations experienced rapid population growth and observed a net rise in the number of people exposed to household air pollution [49]. In Nigeria, for instance, the percentage of the population using solid fuels decreased from 82% to 77%, but due to population growth, the number of exposed individuals still reached 29 million [49]. Similar trends were observed in countries such as Ethiopia and the Democratic Republic of the Congo, where 96% and 93% of the populations, respectively, continue to depend on solid fuels for cooking [49].

Exposure to household air pollution during pregnancy has been associated with increased maternal psychological distress, which in turn can contribute to adverse birth outcomes and gestational ill‑health [67]. Pregnant women using kerosene or firewood in Nigeria had notably higher levels of psychological distress compared with those using ethanol stoves [67]. Although larger cohorts are required to confirm statistically significant links between PM_2.5_ exposure and gestational ill‑health, these findings suggest the need to implement preventive measures to alleviate maternal distress during pregnancy by reducing household air pollution [67]. Studies on household air pollution in Africa emphasize the criticality of implementing evidence‑based policies and making informed decisions promptly to ensure that individuals, including children and adults, residing in Africa have access to better air quality within their households.

#### PM_2.5_ concentrations associated with health outcomes in vulnerable populations in Africa

Throughout any given year, children and infants experience significantly higher risk of exposure to PM_2.5_ pollution compared to adults [68]. This is primarily due to their higher inhalation rate‑to‑body weight ratio, leading to hazard quotients that are approximately three times higher than those of adults [68]. In 2017, a significant proportion of global deaths linked to household air pollution occurred amongst children under the age of five, at about 33% of all such fatalities [65]. The majority of these deaths occurred in Africa, with an estimated half a million deaths (range: 0.35–0.68 million) [65]. PM_2.5_ was found to increase under‑five mortality rates by 2% in Western and Central Africa, while also contributing to a 19% increase in maternal mortality in Central Africa.[69] PM_2.5_ from dust showed a 3% rise in under‑five deaths in Northern Africa, a 1% increase in Western Africa and a significant 10% increase in Central Africa [69]. PM_2.5_ exhibited a substantial effect on under‑five deaths in Western Africa and maternal deaths in Eastern Africa [69]. These findings highlight the significant impacts of ambient PM_2.5_ on under‑five and maternal mortality in Africa, where the exposure levels vary widely and often surpass the WHO’s guidelines [69]. Other vulnerable groups, such as the elderly or individuals with underlying health conditions, are known to have increased susceptibility and higher risk of experiencing adverse health effects in relation to air pollution exposure [68] but African data on these groups are scarce.

Household energy poverty is a pressing challenge faced by urban and peri‑urban communities in SSA and affects mental health outcomes in women [70]. The prevalence of depression varied significantly across communities, with a four‑fold difference observed (36% in Mbalmayo, Cameroon; 20% in Eldoret, Kenya; 9% in Obuasi, Ghana). Women who primarily cooked with charcoal and wood had about 1.5 times higher odds of depression compared with those who primarily used liquefied petroleum gas [70]. Women without access to electricity had 1.4 times higher odds of depression compared with those with access [70]. The prevalence of depression symptoms varies largely across community level socioeconomic gradient and household energy sources, highlighting the complex interplay between energy poverty and mental health.

#### The need for switching to renewable energy in Africa

Energy plays a vital role in driving economic development and serves as the fundamental building block for industrialization [71]. Coal‑based energy generation results in large amounts of air pollution emissions [72] and ought to be responded to with technological advancement. According to a recent study, Africa and African organizations and their partners such as the African Union have the potential to bolster their efforts to adopt renewable energy [73]. Africa could bypass fossil fuel technologies and instead adopt an energy strategy that is climate‑ and environment‑ friendly, fostering low carbon growth whilst meeting developmental needs [73].

Optimal utilization of Africa’s abundant renewable energy resources is crucial for achieving a sustainable energy mix [74]. Effectively harnessing the continent’s vast solar, wind, hydro and geothermal potential can meet the energy demands [74]. Solar power, with its year‑round sunlight, emerges as a viable and cost‑effective option. The mini‑grid strategy, featuring decentralized power distribution networks independent of the national grid, offers a scalable and affordable solution [74]. Integrating various renewable sources with advanced energy storage and smart grid technology, mini‑grids not only provide energy for essential tasks but also foster economic growth and improve social services [74]. Switching to cleaner sources of energy would improve air quality and support climate change mitigation strategies. In cities, in particular, cleaner energy sources would improve the overall air quality such that city‑scale average PM levels can plateau at levels far lower than experienced in Asia.

##### Study limitations

A common challenge amongst public health researchers and data scientists included restricted access to relevant data which was one of the main obstacles encountered in this investigation. Our analysis may not accurately reflect the range of health and air pollution data in Africa since we relied on research articles that are accessible through open‑source literature databases. It is challenging to get a comprehensive picture of the state of air quality throughout the continent because many African nations do not actively participate in scientific study or publication. Future research studies could benefit from direct involvement with governments and other stakeholders in countries with a low scope of research publications to acquire a more comprehensive and accurate picture. Furthermore, although pollutants other than PM_2.5_ may have important consequences for public health, this study only looked at PM_2.5_. The scope of this review did not include other pollutants or important sources of pollution such as automobile and transportation emissions or other pollutants that are not part of PM_2.5_. Although additional research into other pollutants and sources would provide a more thorough assessment of air quality in Africa, our decision to concentrate on one particular pollutant was motivated by the data’s accessibility and its well documented significance to public health.

### Recommendations for improved air quality, human health and economic resiliency

Africa is undergoing economic changes that will continue to expand industrialization and potentially lead to better economic prospects on the continent [75]. Thus, several SSA cities are in economic transition from low to middle/high income status. Effective power infrastructure development in Africa, coupled with sector restructuring, will further spur economic growth, boost electricity generation, promote industrialization, increase incomes, attract investments and avoid reliance on fossil fuel [71]. This urban and economic growth is also expected to result in improved health, quality of life and well‑being of urban dwellers. However, if air quality and climate are not tackled right away, the adverse health impacts from exposures may negate the health advantage of living in cities. Without such transition, air quality will remain a major public health concern and requires systematic and city‑wide measurement programs to improve our understanding of the air quality situation and ensure effective urban air quality management in sprawling cities. Such data will enable Africa to keep track of air quality and its expected adverse impacts on health outcomes. More management programs and research studies are needed to measure air pollutants, determine their sources, and establish air quality targets and their impacts on health [43]. African scientists should collaborate globally to monitor and evaluate air pollution’s impact and develop strategies for better air quality [43].

Policies and measures to reduce air pollution often overlap with those aimed at mitigating climate change. For instance, transitioning from dirty (i.e. fossil fuel, coal, biomass and kerosene) to cleaner energy sources (i.e. solar, wind, geothermal and hydroelectric power), improving energy efficiency and promoting sustainable transportation can simultaneously reduce air pollution and greenhouse gas emissions [76]. It is important to address both air pollution and climate change comprehensively to protect human health, preserve ecosystems and ensure a sustainable future. The interplay between air pollution and climate change also needs to be considered in a systematic manner. Strategies that integrate efforts to mitigate air pollution and combat climate change can yield multiple benefits and contribute to a cleaner and healthier environment.

### Recommendation 1

#### Urbanization and industrialization which continue to increase in Africa should be managed proactively and responsibly (medium to long term)

Urbanization must lead to poverty reduction through service delivery, adequate housing, access to education and employment opportunities. Eliminating poverty reduces air pollution, especially household air pollution and waste burning. African nations are experiencing rapid industrialization due to local economic growth and globalization (e.g. the move of manufacturing by international conglomerates to Africa). This, while an absolute necessity, needs to be coordinated with environmental protection and sustainability, in ways that are harmonized with economic resiliency.

*Actions:*

- Services including electricity generated by renewable energy, water, sanitation and waste removal must be implemented and carried out effectively.
- Urban planning initiatives need to follow smart growth principles to promote environmental protection and sustainability with attention to green space, adequate clean transportation and environment and climate‑conscious zoning systems.
- A well‑developed environmental impact assessment process should be developed and enforced to guide economic expansion.

### Recommendation 2

#### Every African nation state should adopt National Ambient Air Quality Standards (NAAQSs) (short to medium term)

NAAQSs should be adopted with reference to the World Health Organization Air Quality Guidelines and/or Interim Targets together with national ambient air quality monitoring for air quality management, and NAAQS enforcement mechanisms. NAAQSs are based on evidence that shows pollutant concentrations associated with adverse health outcomes, such as the WHO AQGs, and should underpin the threshold concentrations applied in the standards.

*Actions:*

- Harmonize and adapt WHO guidelines to local socio‑economic realities.
- Initiate and/or strengthen air quality management to generate local air quality data along with a dissemination mechanism to the public. In this regard, countries should invest in reference monitors (such as the BAM1022 which continuously monitors ambient air quality. The particulate matter (PM) Monitoring System utilizes the principal of beta ray attenuation to accurately measure and report the concentration of airborne PM in ambient air) against which networks of low‑cost sensors can be calibrated [77]. This example is already being implemented by the Eastern Africa GEOHealth hub, and other countries may need to follow such a successful model [78].
- Use of a network of affordable air quality monitoring systems (e.g. combination of low‑cost sensors, existing monitors, satellite‑based data) along with modern machine learning techniques to integrate data.
- Build capacity for policy enforcement, monitoring and evaluation.

### Recommendation 3

#### Air pollution sources vary and must be identified to develop targeted interventions to tackle and reduce emissions from every source (medium to long term)

Sources of air pollution are electrification specifically from coal‑fired power stations, industry (petrochemical, mining, etc.), vehicles (diesel, ageing vehicle fleets, etc.), household air pollution (dirty fuels used for heating and cooking), illegal waste burning and background dust. There are effective interventions and alternatives to address many sources, and efforts to scale up their implementation are needed.

*Actions:*

- Conduct well‑planned source apportionment studies to understand sources.
- Implement suggested measures that are cost‑effective and proven (known as ‘best buys’). Best buys include cleaner vehicles, affordable public transport, developing better waste management systems, generating less organic waste, reducing open burning, moving to renewable energy and increasing energy efficiency (UNEP) [77].

### Recommendation 4

#### Development of policy actions and enforcement mechanisms should take into account the impacts on vulnerable populations (short to medium term)

It is well known that socio‑economic and cultural drivers put vulnerable populations (e.g. women, children, marginalized communities) at relatively greater risk for adverse health outcomes and economic disadvantages with grave societal consequences. In most African nations, women and children account for the majority of the population. Policy actions on air quality need to have a focus on social justice to protect these vulnerable populations for effective public health and economic resiliency.

*Actions:*

- Map out vulnerable populations (especially marginal communities) within the national, regional and continental context.
- Design intervention programmes to protect vulnerable populations from impacts of environmental hazards in ways that are sustainable and beneficial to the community (e.g. locally‑driven and sustainable alternatives to use of biomass fuel).
- Promote and implement community participation in enforcement of environmentally friendly policy actions.

### Recommendation 5

#### Build capacity for health impact assessments

Fostering collaborations between key institutions and international partners can further bolster expertise and resources, ensuring comprehensive and robust health impact assessments. This collaborative approach not only enhances local capacity but also facilitates knowledge exchange and the adoption of best practices in assessing the health effects of air pollution.

*Actions:*

- Leverage existing well‑defined population demographics and health surveillance to accurately measure health outcomes.
- There is very limited infrastructure and human resources adequately trained to conduct studies that provide evidence to policy makers for appropriate decision‑making. Therefore, there is a need to capitalize universities and other public institutions involved in air quality monitoring to enhance their capability in terms of training and acquisition of instruments.

### Recommendation 6

Research is needed to build evidence foundation for action

*Actions*

We recommend the following research areas to build evidence for action:

- Assessment of spatio‑temporal trends of air quality (along with speciation analysis to understand the potential distinct mix of indoor and outdoor sources in the African context) using data from continuous monitoring of air pollution using high quality monitors, along with those from satellites and networks of low‑cost monitors.
- Assessment of health impacts of air pollution with investment in higher quality health outcomes data based on electronic health records and strategically designed cohort (or intervention) studies.
- Use of state‑of‑the‑art data integration and harmonization techniques to harness currently available data within and across countries to assess the health impacts of air pollution.
- Carrying out policy‑oriented accountability studies to monitor potential health benefits of environmental policies.

## Conclusions

Addressing air pollution prevention remains a vital concern that requires leaders to be ready and willing to lead the public, involve stakeholders and engage other pivotal individuals within a democratic society. Africa occupies a distinctive position, on the verge of an industrial era of progress. African leaders should proactively explore new avenues to integrate non‑polluting renewable energy sources (solar power, wind, hydropower) into the early stages of this development, positioning the continent for a competitive edge in the times to come. Having reviewed the literature on the impacts of air pollution on human health in Africa considering the pressing urbanization and industrialization issues, we presented a set of recommendations and actions that serve as a ‘brain trust’ for African leaders and decision‑makers to consider implementing to address the air pollution/health challenge in Africa. While these recommendations may not be readily implementable, it is essential that Africa strives towards making such positive changes to protect the health and well‑being of its people.

## Additional File

The additional file for this article can be found as follows:
